# Southern Education for Southern Youth

**Published:** 1858-10

**Authors:** 


### "SOUTHERN EDUCATION FOR SOUTHERN YOUTH."

We find upon our table an Address before the Alpha Pi Delta Society of the Cherokee Baptist College, delivered at the commencement, on the 14th of July, 1858, by Hon. W. H. Stiles, of Savannah, Georgia.

The object of this address is to present the importance of educating those who are born and reared, and expect to reside in the South, *at home;* and it would afford us pleasure to publish the whole, if we had space, and it were thought strictly appropriate to a Medical Journal. As it is, however, we shall have to content ourselves with a few extracts. We hope and think that the South is gradually awakening to the importance of this most vital subject.

This classical, sensible, practical and patriotic oration should be read by every true Southerner. We have not only been "hewers of wood and drawers of water" for the North, but ere long, without awaking to a sense of our true condition, we shall be bound hand and foot, and be unable to throw off the yoke. Indeed, now, we awfully fear that it is too late for the South to acquire independence, when there are large numbers in our midst who can calmly contemplate the inevitable fulfillment (without some immediate and heroic effort) of Seward's prophecy, that "It is immaterial whether the North have gained their first victory, or sustained their last defeat."

With the reception of Kansas as a State, at the next session of Congress, will expire, in out judgment, the last hope politically of the South *in the Union.* Well may the people of the North affect now to be less violent and exacting upon the subject of slavery, when they feel that the entire accomplishment of their purposes and designs is only a question of time, if they can only succeed in retaining the Southern States a little longer in the Union ; they well know that traitors are in our midst and continually increasing in numbers, and that soon from division among ourselves we shall be utterly unable to unite in any movement for a separate and independent government.

With the mere remnant of hope left, that the people of the South may yet awake from their sleep of folly, we shall continue to struggle on in our feeble way, to aid in arousing them to a sense of their true condition.

In the name and for the sake of our ancestors, if not for ourselves, if we can do nothing more, let us at least "resolve that from this day forward Southern youths SHALL be educated at Southern Institutions in a Southern land. From this day forward, so far as respects education at least, our motto *shall* be "*Independence now—Independence forever.*"

In conclusion let us commend to our readers a few of the many important and soul-stirring paragraphs with which the address of Mr. Stiles is so replete.

"The institutions of the South and those of the North differ as widely, agreeably at least to the opinion of the latter, as ever did those of Athens and Sparta. The institutions of the one are based upon *slavery*, the institution of the other are based upon what they are pleased to call *freedom.* One section is unalterably identified with *slave* institutions, *slave* property and *slave* labor, the other equally identified with *free* society, *free* labor and even *free* love. The people of the South believe that the prosperity and existence of our Union depend upon the *maintenance* of that institution—the people of the North believe or profess to believe that the prosperity and existence of the Union depend upon its *destruction.* But the inhabitants of the two cities of Greece never committed the folly of subjecting their youth to the instruction of a rival—and shall we, with all the light of the nineteenth century, and all the experience which more than two thousand years can furnish, be guilty of such palpable fatuity? Besides there is still another view in which the folly of the

South is infinitely more glaring. There never existed, so far as history informs us at Athens or Sparta, that deadly animosity against the institutions of the other, as at this day actuates the North against the institutions of the South. While the injury consequently to the Athenian or Spartan from a foreign education, would be confined to the youths themselves, in unfitting them for participation in their native institutions, the injury to Southern youth from a Northern education would not only unfit them for their native institutions, but at the same time implant in them a deadly hostility to the institution which nothing but its destruction could possibly appease.

"But to form a more just comprehension of the evils under which we labor, look for a moment at the practical operation of the system. In the first place, at that most interesting but dangerous period of life, when the principles are still unformed, and the judgment immature, when the mind is most active and the heart most impressible, when in fact the youth is little more than clay in the hands of the potter to be moulded at will 'into a vessel of honor or dishonor,' at that tender period, he is removed from the parental roof and all the salutary infloences of home, and placed under the instruction and subject to the control of those who believe slavery to be *semper et ubique* 'a moral wrong, a violation of the obligations we are under to our fellow-men and a transgression of the laws of our Creator.' * * *

"Nor do the objections to Northern institutions end with abolition instruction and abolition text-books, but applies with equal force to all the intercourse and association of youth. The whole moral atmosphere in the vicinity of these Northern institutions is so highly impregnated as to render it impossible for a student to breathe it without inhaling abolition at every breath. He not only acquires it from his studies, but he imbibes it from the pages of every periodical or the columns of every journal which in his hours of recreation he may chance to take up. He not only hears it discussed every day in the week by all classes and at all hours, but as if there were no 'day of rest' for this wornout and fully exhausted topic, he receives it again on the Sabbath from the sacred desk which has been prostituted to its unholy purposes. In short the contracted hold of a slave-ship upon which these philanthropists are so pleased to descant can not be more mephitic than is the whole moral atmosphere of New-England with the putrescence of this long dead, but still unburied subject. In an atmosphere composed so much of azote and so little of oxygen as to be unable to sustain life, how is it to be expected that a Southern youth exposed to it can survive without inhaling seeds of moral corruption and decay? * * * * * * * * * * *

"We have thus seen not only that slavery has existed in every age and at some period or other in every nation of the earth, but that whenever and wherever it existed, it was accompanied by the highest civilization and greatest prosperity. And we further see that this civilization and this prosperity as invariably deserted them whenever and wherever the institution was abolished. Ought not then an institution which secures these blessings, to be preserved? If so, how is that object to be attained, unless the youths, our future legislators, to whom the government must soon be committed, sustain it. * * * * *

"The drift of opinion at the North is steadily setting to a trial of strength with slavery in its stronghold. The open attack may be deferred a few years longer, but so sure as you are to arrive at manhood if you live, just so sure will that attack come. The area of free-soil is rapidly increasing, ambition and fanaticism are diligently enlarging its borders, the lines of circumvallation are ceaselessly rising around our citadel, already has their chief, from his place in the United States Senate, sent forth to the black columns under his command the cry 'We have hemmed them in, food, water and reinforcements are cut off, the last pass-way of retreat is closed, they must starve, be cut to pieces or surrender.' Study then, my young countrymen, *study* and prepare for the contest.

"Guardians of youthful learning! In your hands rest the destinies of our State. Your dominion is over that germ of all her power the strong and generous and manly spirits of the rising youth around you. It is the lesson of history confirmed by experience, that everything which exalts a nation and renders its institutions permanent, depends upon the character given by education to her boys. Recollect then, that in the great contest which our country seems nearing, it is *mind* which will be in demand, it is the power that comes from *within* which will rule, and this power it is your province to mould. In marshalling forces for the contest, Northern institutions possess no advantage over you, they may, it is true, by more machinery in the way of professors and lectures, contribute to the grace and polish of intellect, but in cultivating and producing the true strength of a nation, *well disciplined minds*—minds habituated to active persevering enquiry and masterly grappling with thought, they should have no superiority. Grace and polish will not avail them in the contest. Robust, symmetrical, well poised minds, alone will prevail. Minds capable of strong, self-moved and independent action, competent to stand alone and defy the world, if God and right demand it, and yet so docile that a child may lead them, if he only hold him by the bands of truth.

It is of immense moment to us in the South that we so educate our youth that they may be able amidst the unparallelled excitements by which they are surrounded, calmly and candidly to appreciate their condition, and suffer themselves neither bound down by the chains of a too rigid conservatism or carried away by the extravagances of 'inevitable progress.' So educate them that they may stand firm and erect amid the storm, yielding neither to the fanaticism of abolition on the one hand, or driven by that madness beyond the constitution into a reopening of an abandoned traffic on the other. So educate them in short, that they may know as well what are the sacrifices of interest and feeling which for the preservation of our Union it may become necessary to make, as what are the obligations to right and honor, they should unhesitatingly maintain even at the cost of a dissolution of this confederacy.

"Friends and patrons of learning who have gathered to witness the exercises of this interesting occasion, a word to you and I have done. If nothing which I have advanced has been able to convince you of the necessity of Southern education for Southern youth, I have in reserve an appeal which the heart of no true Georgirn, I trust, can possibly resist.

"It is the *solemn warning* of our departed sires. It comes to us like "the handwriting on the wall" to the inmates of Belshazzer's palace with all the force of merited retribution. It requires no Daniel to interpret, but stands upon the pages of our statute book, in a language which all may read; that, *'sending them (our youth) abroad to other countries for education will not answer these purposes, is too humiliating an acknowledgement of the ignorance or inferiority of our own, and will always be the cause of so great foreign attachments, that upon the principles of policy, it is inadmissible.'* Not conten with this admonition embraced in the Act of 1785, for the establishment of a State University, our patriot fathers, at the same session of the General Assembly, passed a separate Act disfranchising any person who might be sent abroad for the purposes of education and declaring such ineligible to any office, civil or military, in the State, for a term of years equal to that of their foreign residence. And this statute remains to this day unaltered and unrepealed.

"Have *we*, I would ask, less love for our sons or less regard for our country, than actuated our ancestors? Or is the danger of contamination from a foreign education less now than formerly, less from one obtained in *New* England than from one acquired in *Old* England, less from an education obtained in the abolitionized and inimical States of Massachusetts and Connecticut at the present day than one acquired in the indifferent but friendly countries of Europe in the last century? It will not be admitted! Why then have we, on so vital a subject, for more than seventy-three years, disregarded the wholesome injunctions of that authoritative voice?

Let us pause, before it is forever too late, and gather wisdom from the teachings of the past."

